# Overlapping representations of the neck and whiskers in the rat motor cortex revealed by mapping at different anaesthetic depths

**DOI:** 10.1111/j.1460-9568.2007.05997.x

**Published:** 2008-01

**Authors:** Shashank Tandon, Niranjan Kambi, Neeraj Jain

**Affiliations:** National Brain Research Centre N.H. 8, Manesar, Haryana, 122050, India

**Keywords:** ICMS, intracortical microstimulation, somatosensory, topographic map, vibrissae

## Abstract

The primary motor cortex of mammals has an orderly representation of different body parts. Within the representation of each body part the organization is more complex, with groups of neurons representing movements of a muscle or a group of muscles. In rats, uncertainties continue to exist regarding organization of the primary motor cortex in the whisker and the neck region. Using intracortical microstimulation (ICMS) we show that movements evoked in the whisker and the neck region of the rat motor cortex are highly sensitive to the depth of anaesthesia. At light anaesthetic depth, whisker movements are readily evoked from a large medial region of the motor cortex. Lateral to this is a small region where movements of the neck are evoked. However, in animals under deep anaesthesia whisker movements cannot be evoked. Instead, neck movements are evoked from this region. The neck movement region thus becomes greatly expanded. An analysis of the threshold currents required to evoke movements at different anaesthetic depths reveals that the caudal portion of the whisker region has dual representation, of both the whisker and the neck movements. The results also underline the importance of carefully controlling the depth of anaesthesia during ICMS experiments.

## Introduction

Primary motor cortex, M1, in mammals is located in the frontal lobe rostral to the somatosensory cortex. In one of the early studies Penfield (Penfield & Boldrey, 1937), using surface stimulation, showed that the motor cortex contains an orderly representation, commonly referred to as the homunculus, of the body parts. A similar map has been described in the motor cortex of monkeys (Woolsey *et al*., 1952). The map was later refined using microelectrodes inserted in the depths of cortex to stimulate a small cluster of neurons using trains of electrical pulses by a method that came to be known as intracortical microstimulation (ICMS; e.g. Strick & Preston, 1978; Preuss *et al*., 1996). ICMS has been used to determine the organization of the motor cortex in a wide array of other mammalian species including rats (Hall & Lindholm, 1974; Sanderson *et al*., 1984; Gioanni & Lamarche, 1985; Neafsey *et al*., 1986; Sanes *et al*., 1990). Unlike the precise somatotopy in the somatosensory cortex (Kaas *et al*., 2002), stimulation in the motor cortex evokes movements of a group of muscles that coordinate to generate a natural-like movement for the animal (Schieber, 2001).

Although there is a general consensus on the location and organization of M1 of rats, the extent and boundaries of the representation of movements of different body parts in M1 remains uncertain (Gioanni & Lamarche, 1985; Neafsey *et al*., 1986; Kleim *et al*., 1998). The differences in the published maps are most pronounced in the region representing movements of the mystacial whiskers. These differences could partly stem from the differences in the depth of anaesthesia and the consequent physical state of the animals, such as spontaneous whisking at the time of mapping (Gioanni & Lamarche, 1985; Brecht *et al*., 2004; Haiss & Schwarz, 2005). It is important to establish details of the normal map and to clearly understand the normal organization of the movement map in the rat motor cortex to help compare normal maps to the reorganized maps resulting from injuries or training (Donoghue *et al*., 1990; Sanes *et al*., 1990; Nudo & Milliken, 1996; Kleim *et al*., 1998; Ramanathan *et al*., 2006). In order to address these issues we used ICMS to determine organization of the rat motor cortex at different anaesthetic depths as well as after considering movements evoked at suprathreshold currents. The results have been submitted previously in the form of a brief abstract (Tandon *et al*., 2007).

## Materials and methods

All animal procedures were approved by the National Brain Research Centre Animal Ethics Committee and conformed to the Government of India and NIH guidelines.

### Animal preparation

Fourteen adult Long–Evans or Wistar male rats weighing between 407 and 770 g were used to map the motor cortex. Anaesthesia was induced with an initial dose of 80 mg/kg ketamine hydrochloride and 7 mg/kg xylazine given intramuscularly. Supplemental doses were given to maintain the required anaesthetic levels during the experiment. Nine rats were mapped at light anaesthetic depth and six rats were mapped at deep anaesthesia levels, including one rat that was mapped under both light and deep anaesthesia. Dexamethasone (2 mg/kg i.m.) and glycopyrrolate (6 µg/kg i.m.) were given to reduce swelling of the cortex and to prevent excessive salivation, respectively. The head of the rat was stabilized in a head holder (Kopf, CA, USA) and the cisterna magna was opened to drain out the cerebrospinal fluid. A craniotomy was made to expose the frontal cortex, the dura mater was retracted and a high-resolution photograph of the exposed cortex was taken using a digital camera. The exposed cortex was covered with warm silicon oil to prevent desiccation. Core body temperature was monitored with a rectal probe and maintained at 37 °C with a warm-water blanket placed below the rat. Normal saline was given subcutaneously at regular intervals to keep the animal hydrated.

### ICMS

ICMS was carried out using unipolar parylene-coated tungsten microelectrodes (1 MΩ at 1 kHz, tip diameter 1 µm, shaft diameter 0.127 mm; MicroProbe, MD, USA). The electrode was inserted perpendicular to the cortical surface to a depth of 1200–1500 µm from the surface using a hydraulic microdrive (Kopf). The stimulation parameters were 60-ms trains of 0.2-ms monophasic cathodal pulses (DC) at 300 Hz (Jacobs & Donoghue, 1991; Qi *et al*., 2000). Initially 50 µA current was used to determine whether any movement was evoked. If a movement was visible, the current was progressively decreased until no movement was seen. Threshold current was defined as the minimum current at which a visible movement of any body part was reliably evoked and confirmed by two observers. If no movement was observed at 50 µA, the current was progressively increased until a definite movement was observed and threshold current was determined by decreasing the current. The maximum current used was 100 µA (Neafsey *et al*., 1986) to avoid the possibility of a lesion in the cortex (Asanuma & Arnold, 1975; Neafsey & Sievert, 1982). Initially, for some of the rats we stimulated at currents up to 150 µA to determine whether any additional movements were observed at higher currents. As no major differences were found we restricted the maximum current to 100 µA for the latter experiments. Throughout the experiment the body parts were manually moved intermittently to avoid habituation. The electrode penetration points were marked on an enlarged picture of the brain using surface blood vessels as landmarks, and the threshold currents required to evoke the movements and the type of movements observed were noted. Neurons at a penetration point were considered nonresponsive if no movement was observed. The penetration points were placed in a grid-like fashion at ∼250- to 500-µm intervals, taking care to avoid surface blood vessels. If three or more nonresponsive sites were encountered in a row we returned to a previously responsive part of the cortex and if these points were also nonresponsive the experiment was terminated. Towards the end of the mapping session electrolytic lesions were made by passing cathodal current (10 µA, 10 s) to help align the electrophysiologically obtained map with histological landmarks and the somatotopic map revealed in the sections of the cortex (see below).

### Maintenance of anaesthetic depth

The anaesthetic level was continuously monitored by frequent checking of pinch withdrawal reflex, eyelid reflex and spontaneous rapid whisking of the vibrissae. Animals were said to be under ‘light’ anaesthesia when there was presence of mild spontaneous whisking, and mild pinch withdrawal and eyelid reflexes. The level of anaesthesia was designated ‘deep’ when these reflexes were absent and there were no spontaneous whisking tremors. Supplemental doses of anaesthetics were carefully controlled to maintain the desired anaesthetic depth.

Anaesthetic depth was further characterized by monitoring electrocorticogram (ECoG) from the parietal cortex using a low-impedance (15 kΩ) electrode (Friedberg *et al*., 1999). Power spectra of the ECoG were analysed using Neuroexplorer software (Plexon, TX, USA). The frequency of the most prominent peak at different depths of anaesthesia was correlated with the physical parameters mentioned above.

### Histology

After completion of the recording session, the rats were perfused transcardially with phosphate-buffered saline (0.9% in phosphate buffer, 0.1 m; pH 7.4) followed by 2% paraformaldehyde, and then by 2% paraformaldehyde in 10% sucrose. The brain was removed; the cortex was separated from the underlying tissue and flattened between glass slides. The tissue was cryoprotected overnight in 30% sucrose. The cortex was frozen and cut parallel to the pial surface in 70-µm-thick sections on a sliding microtome. The sections were stained for cytochrome oxidase activity by the procedure of Wong-Riley (1979) to reveal the somatosensory isomorph of the body surface (Dawson & Killackey, 1987; Jain *et al*., 2003). Outlines of the somatosensory isomorph were drawn by overlaying the barrel pattern revealed in the sections from each rat. The electrophysiologically obtained motor map was overlaid on the outline drawing of the histologically revealed chemoarchitecture for each rat using electrolytic lesions as landmarks in Canvas 8 software (Deneba, FL, USA).

### Reconstruction of the cortical maps

For reconstruction of the motor maps the threshold currents required to evoke movements were divided into five groups, 1–20, 21–40, 41–60, 61–80 and 81–100 µA, and the locations were marked accordingly. The locations at which movements were observed at >100 µA are shown separately. The borders between the points were placed to demarcate regions evoking movements of different body parts. The borders were drawn (i) midway between two adjacent sites evoking movements of different body parts, (ii) through the sites where movements of two different body parts were evoked at the threshold current, and (iii) midway between a responsive and a nonresponsive site (Jain *et al*., 1995). We also considered that, as far as possible, regions evoking movements of the same body part fall into a single or minimum number of contiguous zones. Excessive convolutions in the border outline were avoided, if possible, with minor violations of the first and the third conventions mentioned above. Finally the drawings from the histological sections were suitably scaled using microlesions as fiduciary points and aligned with the motor maps.

## Results

We first describe the results demonstrating that anaesthetic depth can be reliably determined based on the criteria used by us. We then show variations in the organization of the motor maps determined at different anaesthetic depths. Finally we show that part of the variation in the motor maps at different anaesthetic depths is due to overlapping representations of the whisker and the neck movement areas.

### Reliability of anaesthetic depth

**Fig. 1:**
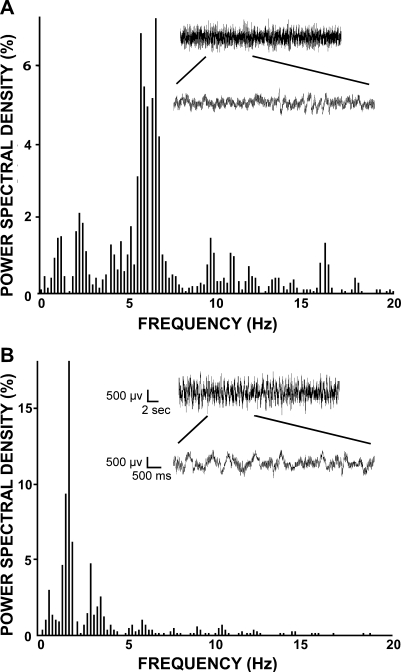
Power spectral densities of ECoG traces from a rat under (A) light and (B) deep anaesthesia. Insets in the graphs show raw ECoG traces along with a part of the trace in an expanded temporal scale. The spectral density was determined in the 0–50 Hz range and the peaks in the 0–20 Hz range are shown. There were no prominent peaks at higher frequencies.

We have classified anaesthetic level as light or deep. The light anaesthesia was characterized by the presence of a pinch withdrawal reflex, eyelid reflex and spontaneous whisking. The deep anaesthetic level was characterized by the absence of all of these physical parameters. In nine additional rats we monitored ECoG from the parietal cortex to establish reliability of determining anaesthetic depth by these physical parameters. The power spectrum of the ECoG showed the most prominent peak at 5–7 Hz in rats that were characterized as lightly anaesthetized based on the physical parameters described above (Fig. 1A). The prominent peak was at 1–2 Hz in rats under deep anaesthesia (Fig. 1B). The power spectrum analysis confirmed that the physical parameters were reliable indicators of anaesthetic depth. In one rat under deep anaesthesia ICMS mapping was carried out while the ECoG was monitored alongside determination of the physical parameters in order to further confirm the reproducibility of the anaesthetic depth (not shown).

We also determined heart rate of all the rats. The rats under deep anaesthesia had lower heart rates (192–216 beats/min) and those under light anaesthesia had higher rates (201–257 beats/min). However, due to large variations in the heart rate between individual rats and the overlap of the heart rate in the two states of anaesthesia it was considered an unreliable indicator of anaesthetic depth.

### Threshold map of the primary motor cortex (M1) at light anaesthetic depth

**Fig. 2:**
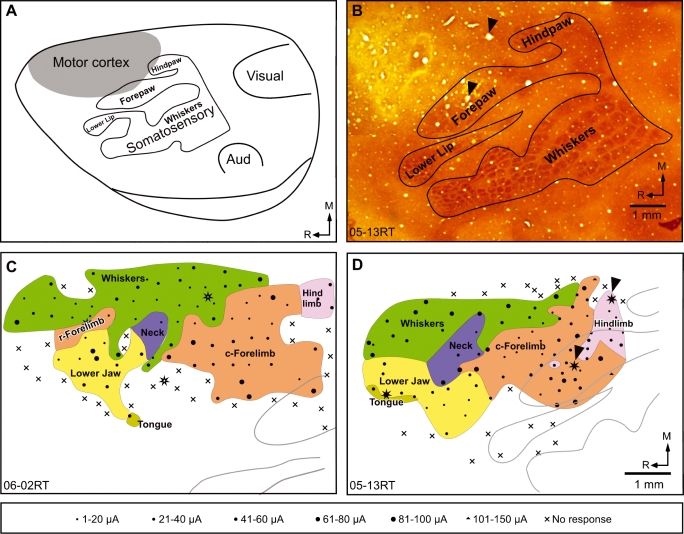
(A) An outline diagram of the dorsolateral view of the cerebral hemisphere of a rat brain showing the location of the motor cortex and its relation to major sensory areas. Body parts in the somatosensory cortex S1 are marked; Aud., primary auditory cortex. (B) A photomontage showing the somatosensory isomorph revealed in sections of the flattened cortex stained for cytochrome oxidase activity, for rat 05–13RT. The location and details of the motor cortical map are described in relation to such somatosensory isomorphs. Arrowheads point to the microlesions used to align it with the motor map shown in (D). (C and D) Organization of the motor cortex in two rats mapped under light anaesthesia. Note the large medial representation of the whiskers and the smaller neck region. The rostral (r) and the caudal (c) forelimb regions are marked. (D) In rat 05–13RT the rostral forelimb was not seen, probably because of low mapping density in the region due to an overlying blood vessel. The grey outlines show portions of the somatosensory isomorph (see A and B). The bottom panel shows the key to the symbols used in C and D. The stars mark the locations of microlesions used to align topographic map to the somatosensory isomorph. R, rostral; M, medial. The scale bar corresponds to the distance on the brain measured during recordings. Scale bar and the orientation arrows shown in D are also valid for C

**Fig. 4:**
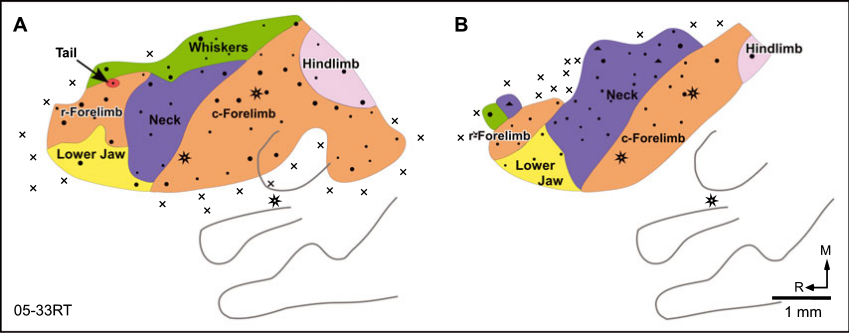
Organization of the motor cortex in a rat mapped (A) first under light and (B) then under deep anaesthesia. Under deep anaesthesia the whisker movements could not be evoked except for one point in the rostralmost region. The neck is greatly expanded, particularly into the caudal whisker region. The caudolateral region of the motor cortex could not be mapped under deep anaesthesia because the cortex became unresponsive due to the length of the experiment. Other conventions as for Fig. 2.

**Fig. 5:**
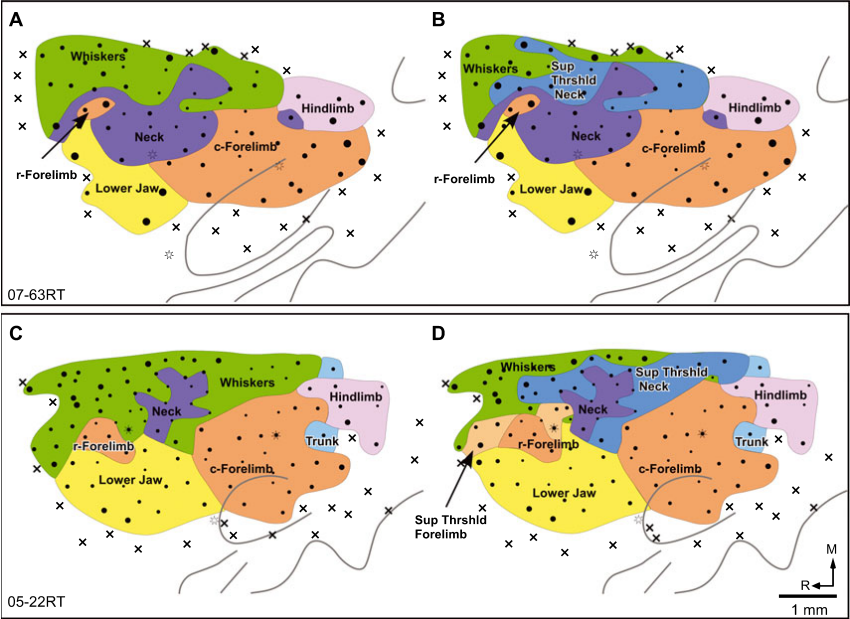
Maps of the motor cortex of two rats at (A and C) threshold currents, and (B and D) when movements at higher currents were considered. Note that at suprathreshold currents the neck movement region expanded into the caudal part of the whisker region. In rat 05–22RT an expansion of the rostral forelimb region is also seen. The whisker movements evoked along with the expanded neck or the rostral forelimb regions are not illustrated in B and D. Sup Thrshld, suprathreshold. Other conventions as for Fig. 2.

Nine rats were mapped at light anaesthetic depth including one rat mapped at both the deep and light anaesthesia levels. Movements were evoked from a region of the frontal cortex just rostral to the primary somatosensory cortex (S1 or the barrel cortex; Fig. 2A and B). The motor cortex extended mediolaterally from the midline to about the lateralmost edge of the somatosensory forepaw region in the barrel cortex. Caudally the movements could be evoked from portions of the cortex that overlapped with the forepaw and the hindpaw regions of the barrel cortex (Fig. 2). No differences were found in the overall organization of the motor cortex between the Wistar and the Long–Evans rats in our studies. For the results described below these strains are considered together. In all the rats we were able to evoke movements of the whiskers, neck muscles, forelimb and lower jaw (Fig. 2C and D; see also Figs 4A and 5A and C). Although the overall topographical organization was similar across the animals, the details of representations of individual body parts varied. The whisker region was located in a medial rostrocaudal strip rostral to the hindpaw representation in the primary somatosensory cortex (S1). Out of a total of 203 sites (for all the nine rats) at which movements of the whiskers were evoked, we mostly observed either retraction of the whole whisker pad (at 92.6% of the sites) or retraction of a group of adjacent whiskers (7.4% of the sites). Single whisker movements were not observed. We did not see the proposed small whisker subregion evoking protraction movements (Sanderson *et al*., 1984; Haiss & Schwarz, 2005) perhaps because of our low mapping density in that region.

The forelimb movement region was located just lateral to the whisker region (Fig. 2C and D; see also Figs 4A and 5A and C). It was rostral and slightly medial to the representation of the forepaw in S1. Movements could also be evoked from parts of the adjacent forepaw representation in S1 where the currents required were generally higher. The forelimb movement region was separated into the rostral and caudal forelimb region by a small portion of the cortex where movements of the neck were evoked. In one rat the rostral forelimb region was not seen (rat 05–13RT, Fig. 2D); this could be due to a gap in the map because of a large blood vessel overlying the cortex just rostral to the neck region. The caudal forelimb region was on average nine times larger than the rostral forelimb region. The most common movement evoked was contralateral elbow flexion (176 or 63% of the total 279 forelimb sites) followed by contralateral wrist extension (82 sites or 29% of the total). Other movements of the contralateral forelimb were finger flexion (five sites), wrist flexion (one site) and wrist abduction (one site). Bilateral movements of the elbow were evoked at 11 sites and the movement of only the ipsilateral elbow was seen at two sites in two rats (05–13RT and 05–06RT). All of these bilateral and ipsilateral movement sites were located in the caudal forelimb region.

The neck region separated the two forelimb regions and occasionally extended medially into the whisker region. The neck movement was evoked from a total of 86 sites in the nine rats. At 76 sites (88.4%) the movement evoked was bilateral, at nine sites (10.5%) it was contralateral and at one site ipsilateral movement was evoked. The movement generally consisted of twitching of the muscles in the back of the neck that were exposed during the procedure to open the fourth ventricle.

The movements of the lower jaw were evoked from a large region lateral to the rostral forelimb and the neck movement regions. The jaw movement region was rostral to the lower jaw and forepaw representations in S1 and occasionally extended up to the S1 forepaw area (see Fig. 5C). The evoked movement was jaw retraction.

Movements of other body parts were also seen in the animals mapped at light anaesthesia. In two rats movements of the eye balls were evoked from a narrow rostrocaudal strip of cortex medial to the whisker representation (not shown). Movements of the tongue were evoked from few sites located lateral to the lower jaw movement region (Fig. 2C and D). At these sites the movement was always tongue retraction and was observed in four rats. Finally, caudal to the whisker and the forelimb regions we could evoke movements of the upper trunk (three rats), hindlimb (eight rats), and tail (one rat). Hindlimb movements included movements of hip, ankle or toes. However, these regions were not mapped in detail and no effort was made to find complete borders surrounding these regions.

### Map of M1 under deep anaesthesia

**Fig. 3:**
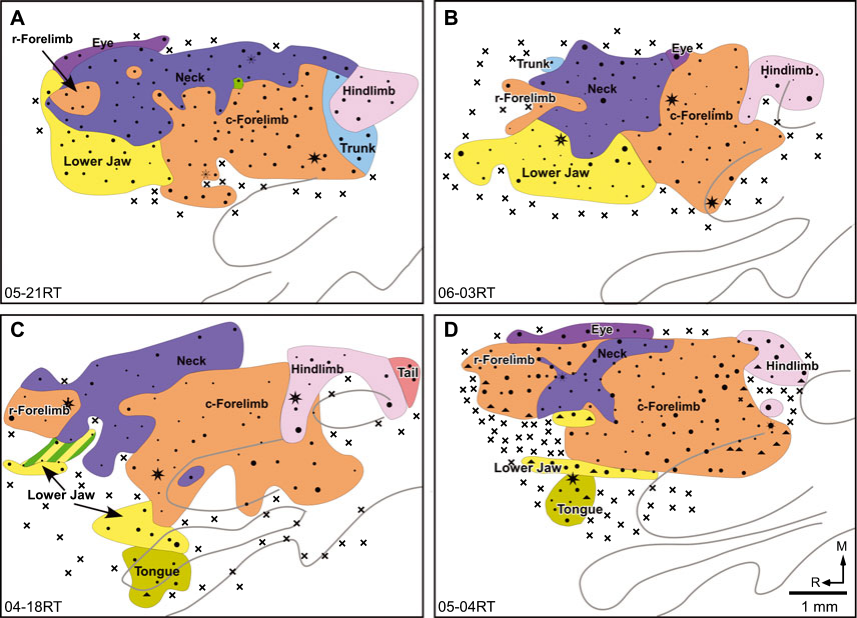
Organization of the motor cortex in four rats (A–D) mapped under deep anaesthesia. Note the absence of the whisker region except for a single point in (A) and two points overlapping the jaw region in (C) The neck representation is enlarged as compared to rats mapped under light anaesthesia (cf. Fig. 2C and D). Other conventions as for Fig. 2.

Six rats were mapped under deep anaesthesia (Fig. 3), including the rat mapped both under light and deep anaesthesia (Fig. 4B). Most of the features of the organization of the motor cortex that were observed in light anaesthesia were also observed in rats mapped under deep anaesthesia. The major difference was that no whisker movements could be evoked in any of the rats under deep anaesthesia (Fig. 3), even when currents as high as 150 µA were tested. Instead, the neck region was greatly expanded into large parts of the region where whisker movements are evoked under light anaesthesia. The neck movement region under deep anaesthesia extended up to the medial eye movement region (Fig. 3A, B and D). Of the total number of 130 sites where neck movement was evoked, at 111 sites (85.4%) it was bilateral neck movement, at 14 sites (10.8%) contralateral neck movement and at five sites only ipsilateral neck movement was observed. Thus the distribution of the nature of movements evoked was not different from that in rats mapped under light anaesthesia.

We determined whether the change in the size of the neck representation could be an artefact of the placement of the ground electrode. In two rats we determined the nature of the movements evoked after relocating the ground electrode from its usual position on the skin of the ipsilateral neck to the skin of the head on the ipsilateral or contralateral side, skin of the neck on the contralateral side or on the pinna. There was no change in the nature of the movement evoked or the threshold currents after changing the position of the ground electrode, confirming that enlargement of the neck representation was not artifactual.

In three of the rats mapped under deep anaesthesia we observed movements of the eyeballs or eyelids, which were evoked from the medialmost strip of the motor cortex (Fig. 3A, B and D). This location was similar to that in the rats mapped under light anaesthesia. However, laterally this region now shared a border with the bilateral neck region.

The rostral and the caudal forelimb regions bordered the bilateral neck region rostrally and caudally. As for the rats mapped under light anaesthesia, the area of the caudal forelimb region was larger than that of the rostral forelimb region. However, the caudal forelimb region was only five times larger than the rostral forelimb area, due to an enlargement in the rostral forelimb area under deep anaesthesia (see below). The percentage of sites in the forelimb representations where fine movements of digits were observed was similar for the rats mapped under light and deep anaesthesia (1.79% of 279 sites for light and 1.5% of 264 sites for deep anaesthesia). The occasional bilateral or ipsilateral movements that were observed under light anaesthesia were not seen at any of the 264 forelimb sites mapped under deep anaesthesia. Lateral to the forelimb and the neck regions we found a region where stimulation evoked movements of the lower jaw.

Other features of the organization of the M1 map such as the regions evoking movements of the tongue (three rats; Fig. 3C and D), upper trunk (one rat; Fig. 3A), hindlimb (hip or ankle, five rats; Fig. 3A–D) and movements of the tail (one rat; Fig. 3C) were observed at locations similar to the rats mapped at light anaesthetic depth. However, these regions were not mapped in detail.

### Dual representation of the neck and whisker areas

We determined whether enlargement of the neck-responsive region in M1 when rats are mapped under deep anaesthesia represents a form of rapid reorganization or it merely reveals a pre-existing neck movement region which was not detected in threshold current maps. Our observations in the rats mapped under light anaesthesia had indicated that, in parts of the whisker representation area, neck movements were evoked at higher currents. In two rats we systematically explored this by noting the movements observed at suprathreshold currents and determined how the suprathreshold map differed from the threshold current map. In both these rats [07–63RT (Fig. 5A and B) and 05–22RT (Fig. 5C and D)], at higher currents movements of the neck muscles along with movements of the whiskers were observed in parts of the motor cortex where only movements of the whiskers were evoked at threshold current. Suprathreshold currents required to evoke movements of the neck in the whisker region were higher by 2–34 µA, except at one site where the neck movement was evoked after the current was increased by 66 µA. Movements of whiskers were not evoked at suprathreshold currents in the neck region. We did not observe similar changes in the boundaries or areas of representations of body parts other than neck even when movements at suprathreshold currents were noted.

**Fig. 6:**
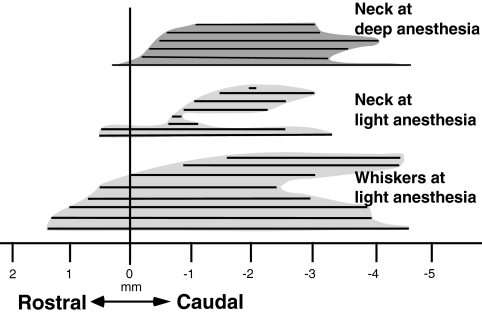
A comparison of the rostrocaudal extent of the whisker and the neck regions mapped under light anaesthesia, and the neck region observed in rats mapped under deep anaesthesia. Each horizontal line depicts the maximum rostrocaudal extent of the representation in a single rat aligned to the rostralmost point of the rostral forelimb region (zero on the horizontal axis). The lines for each representation are sorted top to bottom according to the location of the rostralmost point for that region. Note that most of the expansion of the neck representation in the rats mapped under deep anaesthesia overlaps the caudal part of the whisker representation.

The dual representations of the neck and whisker movements were largely confined to the caudal part of the whisker region (compare maps in Fig. 2 with Fig. 3, and the maps in Fig. 4). Figure 6 shows the rostrocaudal extent of the whisker region and neck region in all the rats mapped under light anaesthesia, and the neck region in rats mapped under deep anaesthesia, illustrating that the rostral whisker region was not replaced by neck representation in rats under deep anaesthesia. Similarly, at suprathreshold currents (Fig. 5), neck movement was not observed in the rostral whisker region.

The observations that (i) only a specific part of the whisker region has dual neck and whisker representation, and (ii) the distribution of the nature of neck movements (in the smaller neck region under light anaesthesia and the larger neck region under deep anaesthesia, or after considering movements at suprathreshold currents) remains the same confirm that the neck and the whiskers have overlapping representations.

### Effect of the depth of anaesthesia on the threshold currents to evoke movements

**Fig. 7:**
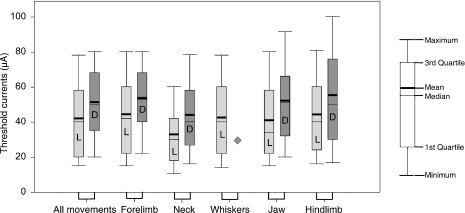
Box plots showing threshold currents required to evoke movements at all the responsive points and for movements of each body part under light and deep anaesthesia. There was significant difference between the median threshold currents under light and deep anaesthesia for all the body parts (*P* = 0.001, except for hindlimb where *P* = 0.041; Mann–Whitney rank-sum test). No whisker movements were evoked under deep anaesthesia (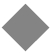). A key to the box plots is shown on the extreme right. L, mapped under light anaesthesia; D, mapped under deep anaesthesia.

We determined the extent to which threshold currents required to evoke movements of different body parts were affected by anaesthetic depth. Overall the movements were evoked at a wide range of overlapping threshold currents at light and deep anaesthetic levels (Fig. 7). There was significant difference (*P* = 0.001; Mann–Whitney rank-sum test) in the median threshold current across all the sites in the motor cortex between the rats mapped at light anaesthetic depth and those under deep anaesthesia. For individual movements of the neck, forelimb, lower jaw and tongue, and hindlimb the current required to evoke movements was also significantly different between the two groups of rats (Mann–Whitney rank-sum test: *P* = 0.001 for neck, forelimb, and lower jaw and tongue, and *P* = 0.041 for hindlimb), indicating that depth of anaesthesia affected the threshold current required to elicit movements. However, the suprathreshold currents that evoked movements of the neck in the whisker region in rats 07–63RT and 05–22RT (Fig. 5), mapped under light anaesthesia, were not significantly different from the threshold currents required to evoke neck movements in rats mapped under deep anaesthesia (*P* = 0.064; Mann–Whitney rank-sum test), suggesting that deep anaesthetic levels reveal a preexisting high-threshold neck region that overlaps the whisker region.

### Areas of representations of body parts

**Fig. 8:**
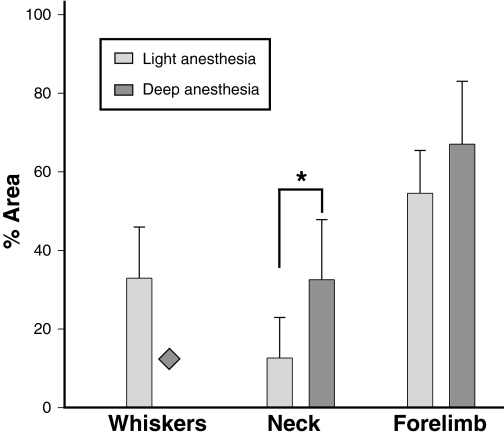
Areas of representation of the whisker, neck and forelimb regions in the motor cortex as a percentage of total area occupied by these representations when the rats were mapped under light and deep anaesthesia. There was no whisker movement under deep anaesthesia (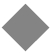). Both the neck and forelimb areas were larger under deep anaesthesia; however, only the areas of the neck representations were significantly different (*P = 0.011; unpaired *t*-test).

We determined relative areas of representations of whisker, neck and forelimb portions of the motor cortex as a percentage of the combined area occupied by these three representations (Fig. 8). In rats mapped at light anaesthetic depth the whisker region occupied 32.9 ± 13%, the neck 12.6 ± 10.3% and forelimb 54.5 ± 10.9% of the total area. In rats mapped under deep anaesthesia the neck region occupied 32.5 ± 15.3%, which was significantly different (*P* = 0.011; unpaired *t*-test) from that under light anaesthesia. The forelimb region was 67 ± 16%, which was more than that under light anaesthesia although not significantly different (*P* = 0.100; unpaired *t*-test). Most of the forelimb region that expanded into the whisker region was the rostral forelimb area (see Figs 3D and 5D). There was an increase of 1.8× in the mean area of rostral forelimb in deep vs. light anaesthesia, while there was no change in the caudal forelimb area. These results indicate that, under deep anaesthesia, most of the nonresponsive whisker region comes to be occupied by the neck movement region and there is some expansion of the rostral forelimb region as well.

## Discussion

The major conclusion of the present study is that parts of the neck and the whisker regions of the motor cortex in rats have overlapping representations. The overlap of representations is revealed by mapping the motor cortex when rats are under deep anaesthesia, as well as by considering movements at suprathreshold stimulating currents.

### Organization of the motor cortex in rats

The overall organization of the motor cortex reported here is similar to that observed by many other investigators (Hall & Lindholm, 1974; Sanderson *et al*., 1984; Gioanni & Lamarche, 1985; Neafsey *et al*., 1986; Sanes *et al*., 1990; Brecht *et al*., 2004). Our maps at light anaesthesia most closely resemble those of Hall & Lindholm (1974) and Sanes *et al*. (1990). As is now generally accepted (Woolsey *et al*., 1952; Neafsey & Sievert, 1982; Sanderson *et al*., 1984; Rouiller *et al*., 1993; see however, Gioanni & Lamarche, 1985), we found dual representation of the forelimb: the rostral forelimb and the caudal forelimb regions separated by the neck representation in all the rats except one. It has been proposed that the rostral region is part of a complete motor map homologous to the premotor (Li *et al*., 1990) or pre-supplementary motor area (Rouiller *et al*., 1993; Wang & Kurata, 1998) of primates. In two rats we observed movements of trunk and tail adjacent to the rostral forelimb region (Figs 3B and 4A), indicating that there might be a complete body map in this region as was proposed based on anatomical connections (Li *et al*., 1990).

We could also elicit movements of the whiskers from a lateral region which largely overlapped the somatosensory whisker region in two of the rats mapped under light anaesthesia (not shown), but not in the rats under deep anaesthesia. Whisker movements from this region have been reported by Gioanni & Lamarche (1985) and Neafsey *et al*., (1986), although others have not reported movements from this region. As reported by Gioanni & Lamarche (1985), we observed more localized movements of whiskers in this region than in the medial whisker region. However, in our preparations stimulation in this lateral whisker zone resulted in movements of a single row of whiskers or the entire whisker pad rather than of individual whiskers. These movements probably reflect direct corticospinal projections from the S1 cortex (Miller, 1987; Li *et al*., 1990).

Considerable variation was seen in the details of the representations across animals. Although relative locations of body parts in the motor map were the same, there were differences in the details of the borders of representations and their relative sizes. Variations in sensory and motor representations across individual subjects has been noted before and might reflect past experiences of individual animals (Jain *et al*., 1998a).

### Dual representations of whiskers and neck

There have been uncertainties regarding representations in the whisker region of the rat motor cortex. A variability in the topography in the whisker region has been illustrated by Neafsey *et al*., (1986). In two different rats (their Fig. 3) they show the medial motor region predominantly evoking movements of either the whiskers or the neck. However, they have not related this variability to the physiological state of the animals. Other investigators have marked this whisker region as the ‘neck/head/whisker’ region (Gioanni & Lamarche, 1985; Kleim *et al*., 1998). Hall & Lindholm (1974) could consistently evoke movements of whiskers from the motor cortex and used this as a test for the state of their preparation. They have not explicitly described the depth of anaesthesia in their sodium pentobarbital-anaesthetized rats. However, the importance of maintaining light anaesthesia to evoke movements of the whiskers was noted by Gioanni & Lamarche (1985). Others have also stressed that whisker movements are evoked only if the rats are lightly anaesthetized and some level of spontaneous whisking is present (Gioanni & Lamarche, 1985; Brecht *et al*., 2004; Haiss & Schwarz, 2005). Our results show that a large part of the whisker region comes to be occupied by the neck movement region in animals that are under deep anaesthesia.

The dual representation seen by us is not similar to the partial overlap of borders of representations of different regions that have been reported earlier in the rat motor cortex (Gioanni & Lamarche, 1985; Kleim *et al*., 1998; Haiss & Schwarz, 2005). We have followed a rigid criterion while demarcating regions by drawing borders through a penetration site where the movements of two body parts were evoked (see also Sanes *et al*., 1990). The only overlap of neighbouring representation that we observed was in one rat in the region of jaw retraction (Fig. 3C). We did not record the electromyogram from the whisker pad muscles and it is possible that there are discharges in the whisker muscles that do not result in any visible movements (Berg & Kleinfeld, 2003) under deep anaesthesia. However, at threshold currents under low anaesthesia there was no movement of the neck muscles in the region of overlap.

A possible substrate for the overlapping neck and whisker representations that were not observed at threshold currents could be long-range horizontal connections. Such connections have been demonstrated between adjacent representations and between neurons with similar response properties (Weiss & Keller, 1994; Matsumura *et al*., 1996; Huntley, 1997; Maynard *et al*., 1999; see Schieber, 2001). Horizontal connections between the neck and the caudal whisker region but excluding the rostral whisker region are possible. Specific and restricted horizontal connections spanning only part of a representation have been shown in the rat motor cortex (Weiss & Keller, 1994; Huntley, 1997).

### Effect of anaesthetics and other physical parameters on the organization of the sensory and motor cortices

Depth of anaesthesia affects the borders and extent of regional representations and the threshold currents required to induce a movement from the motor cortex. In the rat motor cortex, threshold currents required to evoke movements increase with increasing depth of anaesthesia and affect sizes of representations (Sapienza *et al*., 1981; Gioanni & Lamarche, 1985). Anaesthetic depth also modulates receptive fields and sizes of representations in the somatosensory cortex of rats (Erchova *et al*., 2002), and affects modulation of receptive field sizes by nucleus interpolaris (but not principalis) in the ventroposterior medial subdivision of the ventroposterior nucleus (Friedberg *et al*., 1999). In area 3b of owl monkeys, anaesthetics did not result in a change in the size of the receptive fields (Stryker *et al*., 1987; see, however, Duncan *et al*., 1982). Administration of anaesthetic, however, results in a slight decrease in overall representations when measured as a function of blood flow (Chen *et al*., 2005), and affects evoked potentials and EEG (Sloan, 1998; Boisseau *et al*., 2002). Thus anaesthetics might affect the degree of responsiveness of neurons but do not seem to result in any large-scale changes in the topography of the sensorimotor areas or the boundaries of representations. Presently observed changes in the topography in the whisker and the neck region are unlikely to be due to a generalized effect of administration of anaesthetics.

Movements evoked from the rat motor cortex are sensitive to other physical parameters as well. Movements of the whiskers are easier to evoke if the whiskers are held in a protracted position (Gioanni & Lamarche, 1985; see Haiss & Schwarz, 2005), perhaps reflecting the fact that whisker retraction is the most common evoked movement. It is likely that the differences in maps in the whisker region under different depths of anaesthesia reflect differential sensitivities of the movement apparatus of the neck and the whiskers muscles to anaesthesia.

### Plasticity vs. overlapping representations

We regard the emergence of the neck movement region in the whisker region of the rat M1 as overlapping representation rather than a manifestation of anaesthesia-dependent rapid plasticity. The term plasticity is used in many different contexts. One kind of plastic change reflects the normal adaptability of the brain to the situations normally encountered during the life-time. The second kind of plastic changes in the brain organization are seen when the system is subjected to an insult (injury or excessive stimulation that is outside the normal range encountered: Jain *et al*., 1998b; Jain, 2002), and reveal hidden or newly emergent properties of the brain such as due to new growth (Jain *et al*., 2000). Changes in anaesthetic depth reveal neck representation in the whisker region in the threshold map that can be seen under light anaesthesia in animals if additional movements at suprathreshold currents are considered. These changes are thus dependent on experimental conditions and are not true plastic changes. Potential pitfalls of considering only the threshold map as ‘the’ motor map have been discussed before (see Schieber, 2001). Moreover, biophysical mechanisms by which ICMS evokes movements are not completely understood (see Sanderson *et al*., 1984; Schieber, 2001; for a detailed discussion). Threshold maps derived from ICMS provide a convenient way to draw borders of representations and avoid complexities associated with representing a wide array of possible movements. They also provide a method for comparing movement maps across animals and changes in the organization of the map upon deafferentation or other experimental manipulations.

The close association of head movements with whisker retraction could be part of a specific behaviour similar to the behaviourally relevant forearm movements observed following long-train stimulation in rats (Ramanathan *et al*., 2006; see Graziano, 2006). The extensive overlap of the neck and the whisker region perhaps reflects the co-usage of head movement and whiskers for exploratory behaviour.

## Abbreviations

ECoG: electrocorticogram
ICMS: intracortical microstimulation
M1: primary motor cortex
S1: primary somatosensory cortex.
